# Individuals With Pre-arthritic Hip Pain Walk With Hip Motion Alterations Common in Individuals With Hip OA

**DOI:** 10.3389/fspor.2021.719097

**Published:** 2021-08-24

**Authors:** Cara L. Lewis, Anne L. Halverstadt, Kerri A. Graber, Zoe Perkins, Emily Keiser, Hadwin Belcher, Anne Khuu, Kari L. Loverro

**Affiliations:** ^1^Human Adaptation Lab, College of Health and Rehabilitation Sciences: Sargent College, Boston University, Boston, MA, United States; ^2^U.S. Army Combat Capabilities Development Command Soldier Center, Natick, MA, United States

**Keywords:** hip pain, kinematics, gait, pre-arthritic hip disease, dysplasia, femoroacetabular impingement, labral tear

## Abstract

**Background:** Individuals with hip osteoarthritis (OA) commonly walk with less hip extension compared to individuals without hip OA. This alteration is often attributed to walking speed, structural limitation, and/or hip pain. It is unclear if individuals who are at increased risk for future OA (i.e., individuals with pre-arthritic hip disease [PAHD]) also walk with decreased hip extension.

**Objectives:** (1) Determine if individuals with PAHD exhibit less hip extension compared to individuals without hip pain during walking, and (2) investigate potential reasons for these motion alterations.

**Methods:** Adolescent and adult individuals with PAHD and healthy controls without hip pain were recruited for the study. Kinematic data were collected while walking on a treadmill at three walking speeds: preferred, fast (25% faster than preferred), and prescribed (1.25 m/s). Peak hip extension, peak hip flexion, and hip excursion were calculated for each speed. Linear regression analyses were used to examine the effects of group, sex, side, and their interactions.

**Results:** Individuals with PAHD had 2.9° less peak hip extension compared to individuals in the Control group (*p* = 0.014) when walking at their preferred speed. At the prescribed speed, the PAHD group walked with 2.7° less hip extension than the Control group (*p* = 0.022). Given the persistence of the finding despite walking at the same speed, differences in preferred speed are unlikely the reason for the reduced hip extension. At the fast speed, both groups increased their hip extension, hip flexion, and hip excursion by similar amounts. Hip extension was less in the PAHD group compared to the Control group (*p* = 0.008) with no significant *group-by-task* interaction (*p* = 0.206). Within the PAHD group, hip angles and excursions were similar between individuals reporting pain and individuals reporting no pain.

**Conclusions:** The results of this study indicate that kinematic alterations common in individuals with hip OA exist early in the continuum of hip disease and are present in individuals with PAHD. The reduced hip extension during walking is not explained by speed, structural limitation, or current pain.

## Introduction

Hip osteoarthritis (OA) will affect one in four individuals in their lifetime (Murphy et al., 2010). Individuals with hip OA have impaired physical function and decreased physical activity (Judd et al., 2014). The disability associated with hip OA is not an issue of age or chronicity alone. Two-thirds of young adults with recently diagnosed hip OA (ages 20–55) reported significant OA-related work disability and nearly a 40% reduction in quality of life compared to population normative values (Ackerman et al., 2015). Given the substantial impact of hip OA on function and quality of life, an improved understanding of early, potentially modifiable, factors is imperative.

Individuals with hip OA commonly walk with less hip extension compared to individuals without hip OA (Ornetti et al., 2011; Eitzen et al., 2012; Rutherford et al., 2015; Constantinou et al., 2017; Farkas et al., 2019). Decreased hip extension is one of the highest discriminatory gait features of hip OA (Meyer et al., 2015). If not compensated for by increasing peak hip flexion, reduced hip extension may contribute to a reduction in hip excursion during walking as well (Eitzen et al., 2012; Foucher, 2017). The decreased hip extension and excursion noted in individuals with hip OA is often attributed to three potential factors: walking speed, structural limitation, and/or hip pain. Individuals with hip OA often have a slower preferred walking speed than individuals without hip pain (Constantinou et al., 2014, 2017), and a slower walking speed would require less hip extension and excursion. However, the finding of reduced hip motion in individuals with hip OA often persists after statistically adjusting for speed (Foucher et al., 2007; Eitzen et al., 2012), suggesting that reduced hip motion is a consequence of altered mechanics, not walking speed. Hip motion during walking nears the end range of hip extension. Reduced hip extension due to a structural limitation would imply that individuals with hip OA are near or at the end of their available hip extension, and thus are unable to extend the hip more while walking. Given that sagittal plane hip excursion during gait decreases as the severity of structural hip OA worsens (Rutherford et al., 2015), this explanation seems plausible. Alternatively, individuals may intentionally limit their hip extension during walking as a learned behavior, potentially in response to pain. The persistence of this gait modification following total hip arthroplasty (Foucher et al., 2007; Bennett et al., 2008) would support this assertion of a learned behavior. A combination of these factors likely contributes to the decreased hip extension noted in individuals with established hip OA, making it difficult to determine the primary source of this movement alteration.

Kinematic alterations are theorized to alter joint loading and contribute to degeneration of articular cartilage and progression of OA and/or pain (Hodges and Tucker, 2011; Felson, 2013). In individuals with hip OA, increased peak hip flexion during early stance has been associated with an increased risk of worsening hip structure (Kumar et al., 2018). In this same study, those with worsening hip structure also had less hip extension, although not significantly so. The presence of structural damage is not tightly linked to hip pain (Birrell et al., 2005; Heerey et al., 2018; Park et al., 2021). Worsening pain has been associated with limited hip extension and external rotation during walking in females with mild to moderate hip OA (Tateuchi et al., 2019). Together, these findings suggest that kinematic alterations may contribute to the development of hip OA or pain, and may be a modifiable factor to target for prevention if detected early enough.

Hip dysplasia and cam morphology are two structural variants in hip morphology which increase the risk of developing future hip OA (Ganz et al., 2003; Agricola et al., 2013; Thomas et al., 2014; Nelson et al., 2016; Saberi Hosnijeh et al., 2017; Wyles et al., 2017). In hip dysplasia, there is decreased coverage from the acetabulum over the femoral head, as commonly measured as a decreased center-edge angle. Cam morphology, an asphericity of the femoral head, is measured as an increased alpha angle. Individuals with cam morphology, hip pain, and clinical signs including positive provocative tests are classified as having femoroacetabular impingement (FAI) syndrome (Griffin et al., 2016). Degeneration of the acetabular labrum, either with or without structural variation, also contributes to the risk of cartilage degeneration and OA (Altenberg, 1977; McCarthy et al., 2001; Lewis and Sahrmann, 2006). Thus, these intra-articular hip conditions, when painful, are often referred to as pre-arthritic hip disease (PAHD).

Similar to hip OA, decreased peak hip extension has been noted in studies of individuals with PAHD (Romano et al., 1996; Jacobsen et al., 2013; Skalshøi et al., 2015; King et al., 2018; Lewis et al., 2018a) while other studies found no difference (Pederson et al., 2004; Savage et al., 2021). Again, it is unclear if the observed reduction in hip extension is due to walking speed, structural limitation, or pain. Investigating hip motion during walking in individuals at risk for future development of hip OA may provide insight into factors contributing to the kinematic changes noted once OA is established. While altering bony hip structure requires surgical intervention, identifying early modifiable risk factors such as altered gait mechanics could inform interventions to reduce or delay the onset and progression of hip OA in individuals with elevated risk.

Therefore, the purpose of this study was to determine if hip motion alterations common in individuals with established hip OA are also present in individuals with PAHD compared to individuals without hip pain during walking, and to investigate potential reasons for these alterations. We assessed sagittal plane hip kinematics in individuals with PAHD and individuals without pain walking at three speeds; one speed was consistent for all participants (1.25 m/s) while the other two were based on the individual's preferred overground walking speed. We first hypothesized that individuals with PAHD would have decreased peak hip extension compared to individuals without hip pain when walking at their preferred speed. Assuming that the decreased hip extension would be due to slower walking speed, we hypothesized that no group difference would exist when all individuals walked at the same prescribed speed. We also hypothesized that individuals with PAHD would not increase their hip extension as much as the Control group when walking at a speed 25% faster than their preferred walking speed. If confirmed, this would suggest that structural limitations reduce hip extension during walking. Finally, assuming that the reduced hip extension was related to pain during walking, we hypothesized that individuals with PAHD who reported hip pain during the walking task would walk with less hip extension than individuals with PAHD who reported no pain during the task.

## Materials and Methods

### Study Design

This was a cross-sectional study conducted in a university research laboratory setting. This study was approved by the Institutional Review Boards of Boston University and Boston Children's Hospital and all individuals provided written informed consent prior to participation. Data from some of the participants included in this study have been published elsewhere (Lewis et al., 2018a,b; Loverro et al., 2019).

### Participants

This study capitalizes on data collected as part of ongoing studies investigating the interaction of movement and hip morphology in young and middle-aged adults with and without hip pain. To be included in the studies, individuals had to be between 14 and 50 years of age and able to walk safely for 10 min without assistance. Individuals with a history of neurological disorder, history of back surgery, or current back, knee, or ankle pain were excluded from both groups.

We recruited individuals with hip pain and diagnosed dysplasia, FAI syndrome, and/or a labral tear through local orthopedic clinics, and individuals seeking care for hip pain through local rehabilitation clinics. We also included individuals who contacted our research staff directly.

To be included in the hip pain group, individuals had to have their pain reproduced by at least one of three provocative tests performed during the study visit. The three provocative tests, which are sensitive for intra-articular hip pathology (Clohisy et al., 2008; Maslowski et al., 2010), included (1) flexion, adduction, internal rotation (FADIR) test; (2) flexion, abduction, external rotation (FABER) test; and (3) resisted straight leg raise. For the FADIR test, which has also been called the anterior impingement test, the hip was passively flexed to 90°, and then adducted and internally rotated (Ganz et al., 2003). For the FABER test, the hip was passively positioned in flexion, abduction, and external rotation with the foot of the tested leg on top of the contralateral knee (Troelsen et al., 2009). For the resisted straight leg raise (Stinchfield test), the leg was passively positioned in 30° of hip flexion with the knee extended (Maslowski et al., 2010). The participant was then asked to keep the leg in that position without assistance, and continue to hold the position as resistance was applied at the distal leg. If the test reproduced the individual's pain, the test was considered positive.

In the hip pain group, we excluded individuals who reported trauma (accident and major fall) as the precipitating incident of their pain. We did not exclude individuals who had previous hip surgery. Studies suggest that walking patterns change minimally, if at all, following hip surgery for FAI syndrome (Rylander et al., 2011; King et al., 2018).

We recruited a convenience sample of individuals without hip pain through flyers and postings around the university campus, and through word of mouth. Exclusion criteria for this Control group included current or recent (within the last 2 months) lower extremity injury, history of lower extremity orthopedic surgery, history of hip pain, and hip or groin pain or discomfort during any of the provocative tests performed during the study visit.

### Instrumentation

We recorded whole body kinematic data of the trunk, pelvis, and lower extremities using a 10-camera motion capture system (Vicon Motion Systems Ltd. Centennial, CO, USA) sampling at 100 Hz. Participants walked on an instrumented split-belt treadmill (Bertec Corp, Columbus, OH, USA) sampling at 1,000 Hz to allow for continuous collection of gait kinematics. Retro-reflective markers were placed over 30 bony landmarks on the trunk and pelvis and bilaterally on the lower extremities as previously described (Lewis et al., 2015). An additional four rigid clusters that each contained four, non-collinear markers each were positioned over the thighs and shanks and attached via neoprene wraps and hook and loop fasteners (Cappozzo et al., 1997).

### Self-Reported Outcome Questionnaires

Participants completed a set of self-report questionnaires commonly used in the PAHD literature (Kennedy et al., 2009; Lamontagne et al., 2009; Harris-Hayes et al., 2013; Hunt et al., 2013; Samaan et al., 2017). These included the UCLA activity score (Amstutz et al., 1984), the modified Harris Hip Score (mHHS) (Byrd and Jones, 2000), and the hip disability and osteoarthritis outcome score (HOOS) with subscale scores (Klässbo et al., 2003). The Western Ontario and McMaster Universities Osteoarthritis Index (WOMAC) was scored from the HOOS since it contains all the WOMAC questions (Nilsdotter et al., 2003). The mHHS scores, after multiplying by 1.1 to convert from 91 points to 100 points, can be interpreted as 90 to 100, excellent; 80 to 89, good; 70 to 79, fair; and below 70, poor (Byrd and Jones, 2000). Beginning in 2013, participants also completed the three level version of the EuroQol-5 Dimension Questionnaire (EQ-5D) (EuroQol Group, 1990; Brooks, 1996; Shaw et al., 2005) and the Short Form Health Survey (SF-12) (Ware et al., 1996) to capture overall quality of life (Guyatt et al., 1993). The EQ-5D includes the index, which ranges from −0.109 (worst) to 1.0 (best), and the visual analog scale (VAS) for current health status, which ranges from 0 (worst) to 100 (best). The SF-12 allows for determination of a Physical Component Summary and a Mental Component Summary.

### Experimental Protocol

For testing, all participants wore a tight-fitting shirt, spandex shorts, and their own exercise shoes. Prior to data collection, the three provocative hip tests were performed on each participant. Preferred walking speed was determined by taking the average time of five five-meter trials as participants continually walked laps around the lab. We then placed reflective markers on the participant, as described above. After marker placement, we collected a static standing calibration trial with the participant standing in a neutral posture. Joint centers for the hips and knees were created using this trial. We removed the medial knee and ankle markers after this trial so they did not interfere with walking.

Participants completed at least 90 s of continuous walking on a split-belt treadmill resulting in a minimum of ~80 strides at each of three speeds: a self-selected preferred speed, a fast speed that was 25% faster than the preferred speed, and a prescribed speed of 1.25 m/s. The preferred speed was always collected first to capture the individual's natural walking pattern before enforcing the speed constraints, followed by the fast speed, and finally the prescribed speed. As walking speed affects gait kinematics (Crowinshield et al., 1978; Crosbie et al., 1997; Lelas et al., 2003; Bejek et al., 2006; Chung and Wang, 2010), the prescribed speed condition allowed us to compare kinematics when all participants walked at the same speed.

Every 30 s during each walking task, each participant was asked to verbally rate his or her pain on an 11-point numeric rating scale (NRS) with 0 being no pain and 10 being extreme pain (Downie et al., 1978). Pain data were dichotomized, similar to Savage et al. (2021), to separate those reporting pain during the walking task (average NRS during the task > 0) from those reporting no pain (NRS = 0). This dichotomization was done at the task level.

### Data Analysis

Marker trajectory data were labeled and gaps were filled using Vicon Nexus (Vicon Motion Systems Ltd, Centennial, CO, USA). Marker trajectories and ground reaction force data were filtered using a low-pass fourth-order Butterworth filter with a cutoff frequency of 6 Hz and 10 Hz, respectively. Visual3D (C-Motion, Inc., Germantown, MD, USA) was used to create an eight-segment hybrid model for each participant. The CODA pelvis model was used to define the pelvis and the hip joint centers (Bell et al., 1989). For this analysis, we used Visual3D to calculate kinematics of the hip from marker trajectories. Pelvic segment angles were defined with respect to the laboratory coordinate system. Hip joint angles were defined as the angle between the thigh and pelvis segments using a Cardan X-Y-Z (mediolateral, anteroposterior, vertical) rotation sequence (Cole et al., 1993). Ground reaction force data were used to determine heel strike to allow normalization to the gait cycle (heel strike to ipsilateral heel strike).

For each stride, hip and pelvic angles were normalized to the gait cycle and exported for further analysis. We used a custom MATLAB program (Mathworks Inc., Natick, Ma, USA) to extract the dependent variables of interest which included peak hip angles and hip excursion in the sagittal plane. The mean peak angles across all strides for each side were used for statistical analysis.

### Statistical Analysis

The dependent variables for all analyses were peak hip extension, peak hip flexion, and hip excursion during walking. First, we used a linear regression analysis, with group (PAHD, Control) and sex (female, male) as between-participant factors and side (more painful hip, less pain hip) as the within-participant factor to test for differences in sagittal plane hip kinematics during walking. For participants with PAHD, the more painful side was determined by self-report; for the Control group, the more painful side was randomly assigned. We included three interactions in the model: *group-by-sex, group-by-side*, and *group-by-sex-by-side*. As each hip was included in the analysis and the groups were of unequal sizes, a generalized estimating equation (GEE) correction was applied to the linear regression model. The GEE approach is similar to the more commonly used repeated measures Analysis of Variance but has higher power and is more robust (Ma et al., 2012). The analysis was first performed on data from the preferred walking speed task as is commonly done in studies of PAHD and hip OA. The analysis was then repeated for the prescribed speed task to investigate if any differences noted at the preferred speed were no longer present when all participants walked at the same speed. To understand if individuals with PAHD were able to increase their hip extension and excursion similarly to individuals without hip pain, we repeated the analysis including data from the preferred and fast walking speed tasks, and added task as a main effect and interaction term. Finally, to investigate if the limited hip extension was due to hip pain, we performed the same linear regression analyses with GEE correction including only the PAHD group. In this analysis, we compared those who reported pain during the walking task to those who reported no pain during the task.

All analyses were run in IBM SPSS Statistics version 24 (IBM Corporation, Armonk, NY). The alpha level was 0.05 and least significance difference (LSD) pairwise comparisons were performed if the group effect was significant (*p* < 0.05). For any significant interactions, only pre-planned analyses were conducted. For interactions involving sex, only within-sex comparisons were analyzed. For interactions involving group and task, only comparisons between groups were analyzed. Although our hypotheses were directional, we used two-tailed analyses to be conservative.

To explore the data beyond the typical focus on discrete peak angles, we used statistical parametric mapping (SPM) (Friston et al., 2007) to analyze the one-dimensional time series data, normalized from heel strike to ipsilateral heel strike. SPM uses random field theory to make statistical inferences about the likelihood of observed differences between curves occurring by chance. Based on the smoothness of the curves, a threshold is determined. The threshold is determined based on the value beyond which less than 5% of the data would be expected to reach by random chance. The regions where the difference between the curves exceeds this threshold (“supra-threshold clusters”) are considered statistically significant. All SPM analyses were connected using the open-source spm1d code (v.M.0.4.7, www.spm1d.org) in MATLAB (R2020b, 9.9.0.1524771, The MathWorks Inc, Natick, MA). For this analysis, we used a general linear model accounting for sex (dichotomous) and, for the preferred and fast trials, walking speed (linear).

## Results

### Participants

**Figure 1:**
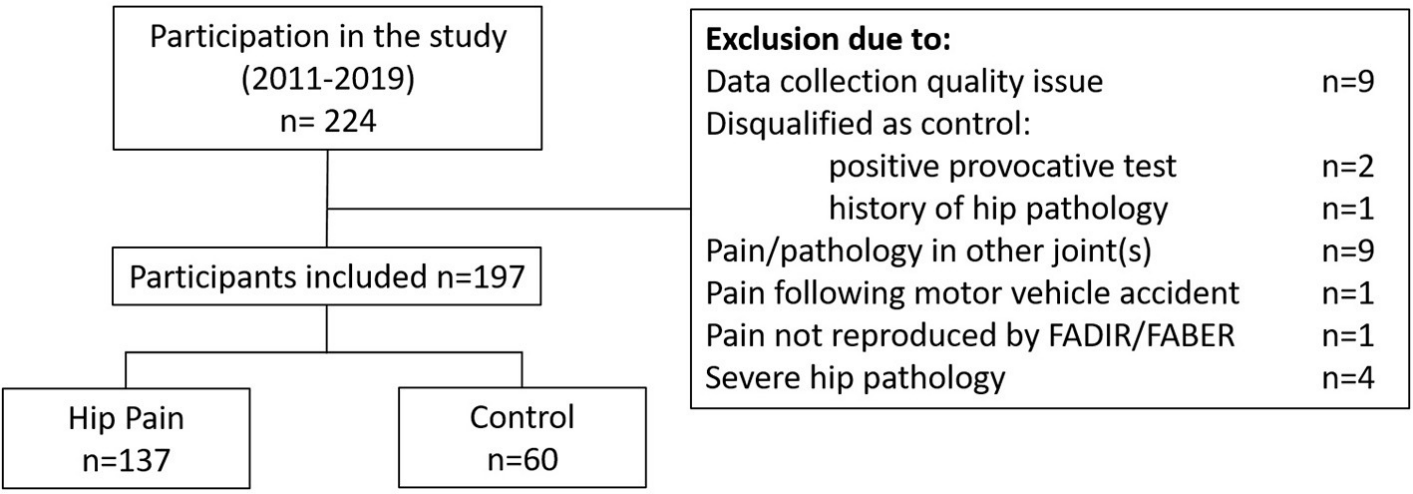
Participant flow diagram.

The study included data from 197 individuals out of the 224 individuals who participated between June, 2011 and March, 2020 (Table 1). Twenty-seven individuals were excluded from this analysis (Figure 1); issues with motion capture data quality and pain or pathology in joints other than the hip were the primary reasons for exclusion. The PAHD group included 137 individuals while the Control group included 60 individuals. Groups were not different in terms of height (*p* = 0.99), mass (*p* = 0.22), BMI (*p*= 0.06) or UCLA activity level (*p* = 0.53). The PAHD group was, on average, older than the Control group (*p* = 0.003); both groups spanned a large age range (PAHD: 14–49 years; Control: 18–46).

**Table 1:** Participant characteristics.

	**PAHD**		**Control**	
	**Female** **(***n*** = 100)**	**Male** **(***n*** = 37)**	**Female** **(***n*** = 35)**	**Male** **(***n*** = 25)**
**Demographic data** ^*^				
Age, y	27.4 ± 8.6	27.9 ± 9.5	23.8 ± 5.4	23.6 ± 5.6
Height, m	1.66 ± 0.07	1.81 ± 0.07	1.64 ± 0.07	1.79 ± 0.07
Mass, kg	66.6 ± 10.2	80.7 ± 10.7	61.3 ± 8.4	77.5 ± 12.4
BMI, kg/m^2^	24.1 ± 3.4	24.7 ± 3.1	22.8 ± 2.5	24.1 ± 2.9
UCLA activity score (0-10)^†^	7.8 (2–10)	8.3 (4–10)	8.4 (4–10)	8.6 (5–10)
Preferred walking speed, m/s	1.29 ± 0.18	1.23 ± 0.17	1.29 ± 0.16	1.26 ± 0.19

*PAHD, pre-arthritic hip disease; BMI, body mass index; UCLA, university of California at Los Angeles*.

* *Values are mean ± SD and analyzed with independent-samples t tests unless otherwise indicated*.

† *Values are median (range) and analyzed with the Mann-Whitney U test. Data were missing for 1 female and 1 male with PAHD and 2 female Control participants*.

As expected, the PAHD group had significantly worse scores than the Control group (*p* < 0.001) on all hip-focused patient reported measures (Table 2). For the mHHS, all individuals in the Control group were categorized as “excellent”; in the PAHD group, 15 (10.9%) were excellent, 41 (29.9%) good, 29 (21.2%) fair, and 48 (35.0%) poor. The wide range of mHHS scores in the PAHD group (44 to 100) highlights the heterogeneity of the hip pain experience.

**Table 2:** Self-reported measures of function and quality of life.

	**PAHD**		**Control**	
**Questionnaire**	**Females** **(***n*** = 99)**	**Males** **(***n*** = 36)**	**Females** **(***n*** = 34)**	**Males** **(***n*** = 26)**
**Self-reported measures** ^*^				
mHHS (0–100)^†^	73.3 ± 13.3	77.4 ± 11.1	100.1 ± 0.2	99.9 ± 0.9
HOOS subscales^‡^				
Pain (0–100)	69.6 ± 17.5	71.3 ± 16.1	99.9 ± 0.9	99.7 ± 1.5
Symptoms (0–100)	68.2 ± 18.0	68.6 ± 16.8	96.3 ± 7.0	98.5 ± 3.7
Functional activities (ADL) (0–100)	83.2 ± 16.1	85.4 ± 13.8	100.0 ± 0.0	99.9 ± 0.3
Recreation/sport activities (0–100)	63.6 ± 23.3	68.8 ± 21.5	99.8 ± 1.1	99.8 ± 1.2
Quality of life (0–100)	47.0 ± 22.7	44.4 ± 21.9	99.4 ± 2.4	99.5 ± 2.5
**WOMAC subscales** ^**‡**^				
Pain (0–20)	5.2 ± 3.6	4.9 ± 3.3	0.1 ± 0.3	0.0 ± 0.0
Stiffness (0–8)	2.4 ± 1.7	2.2 ± 1.3	0.1 ± 0.4	0.0 ± 0.2
Function (0–64)	11.4 ± 11.0	9.9 ± 9.4	0.0 ± 0.0	0.0 ± 0.2
EQ-5D (0–1.00)^§^	0.78 ± 0.14	0.78 ± 0.12	0.96 ± 0.12	1.00 ± 0.00
EQ VAS (0–100)^∧^	77.3 ± 18.0	77.8 ± 13.6	87.7 ± 6.7	86.7 ± 6.5
SF-12 PCS^§^	47.8 ± 9.8	48.3 ± 10.0	59.2 ± 2.5	57.7 ± 3.8
SF-12 MCS^§^	51.8 ± 11.1	51.1 ± 11.6	50.1 ± 8.0	52.5 ± 6.4

*PAHD, pre-arthritic hip disease; HOOS, hip disability and osteoarthritis outcome score; mHHS, modified harris hip score; WOMAC, western ontario and mcmaster universities osteoarthritis index; EQ-5D, EuroQoL 5 dimension questionnaire; EQ VAS, EuroQol visual analog scales; SF-12 PCS, 12-Item short form survey physical component score; SF-12 MCS, 12-item short form survey mental component score*.

* *Values are mean ± SD and analyzed with independent-samples t tests unless otherwise indicated*.

† *Values were missing for 3 females and 1 male with PAHD and 2 female Control participants*.

‡ *Values were missing for 1 female and 1 male with PAHD and 1 female Control participant*.

§ *Values were missing for 7 females and 1 male with PAHD and 12 female and 7 male Control participants*.

∧ *Values were missing for 6 females and 3 males with PAHD and 11 female and 7 male Control participants*.

Not only did individuals with PAHD have worse hip-specific scores, the generic quality of life scores were also lower than the Control group. Compared to the Control group, the PAHD had lower EQ-5D index value (mean difference: 0.198; 95% CI: 0.162, 0.235, *p* < 0.001) and VAS health status score (mean difference: 10; 95% CI: 6.4, 13.55, *p* < 0.001). The Physical Component Summary score of the SF-12 was also 10.5 points lower (95% CI: 7.5, 13.7, *p* < 0.001) in the PAHD group compared to the Control group. Despite lower scores on these measures, the groups were not different for the Mental Component Summary of the SF-12 (mean difference: 0.5; 95% CI −2.5, 3.5; *p* = 0.758) suggesting that in our PAHD group physical health was more impacted than mental health.

### Effect of Sex

**Figure 2:**
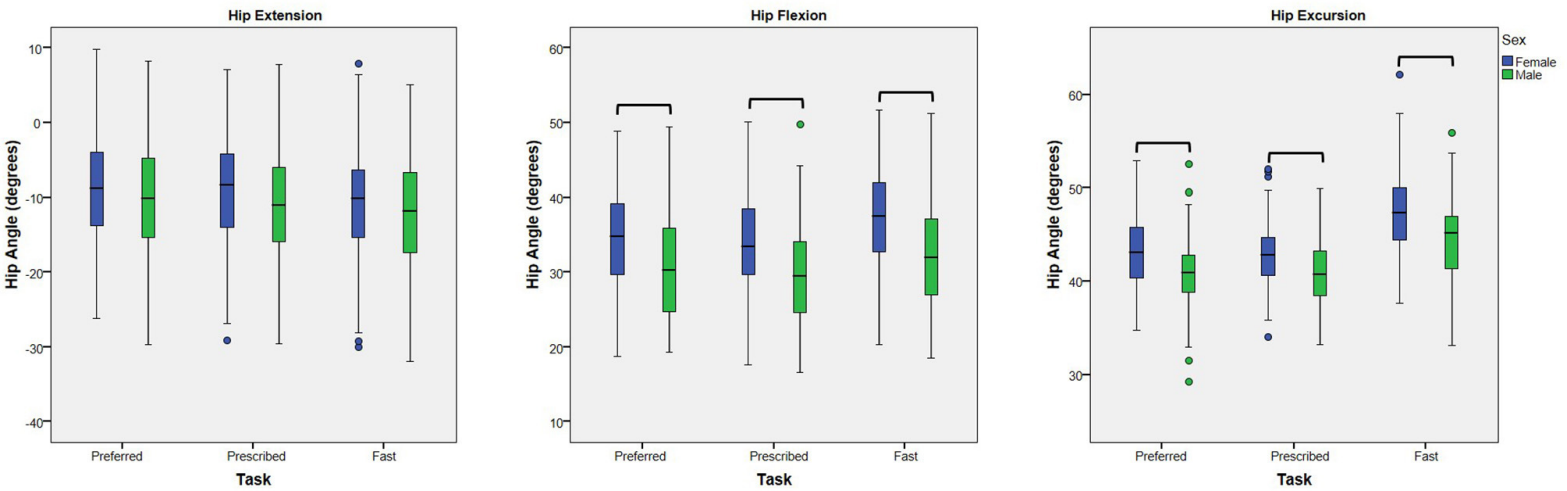
Effect of Sex: Box plots (and outliers) for each dependent variable (peak hip extension, peak hip flexion, and hip excursion) for females and males walking at each of three speed tasks (preferred, prescribed, and fast). At each walking speed, males walked with less hip flexion and less hip excursion than females. No differences in peak hip extension were noted. Hip flexion is represented as positive. Bars indicate significant differences.

A main effect of sex was noted for peak hip flexion and hip excursion for all analyses (*p* < 0.001), but not for peak hip extension (*p* ≥ 0.095) (Table 3). At the preferred speed, males walked with 3.9° less hip flexion (95% CI: 1.7, 6.1°) and 2.5° less excursion (95% CI: 1.2, 3.7°) than females. At the prescribed speed, males walked with 4.1° less hip flexion (95% CI: 1.9, 6.2°) and 2.1° less hip excursion (95% CI: 1.1, 3.1°) than females (Figure 2). At the fast speed, males walked with 4.7° less hip flexion (95% CI: 2.4, 7.0°) and 3.1° less excursion (95% CI: 1.7, 4.5°) than females.

**Table 3:** Results of linear regression with generalized estimating equation correction for each dependent variable (hip extension, flexion, and excursion) at the self-selected preferred walking speed and the prescribed (1.25 m/s) walking speed.

		**Preferred**						**Prescribed**					
**Variable**		**Extension**		**Flexion**		**Excursion**		**Extension**		**Flexion**		**Excursion**	
	**df**	**Wald** **Chi-** **Square**	***p*** **-value**	**Wald** **Chi-** **Square**	***p*** **-value**	**Wald** **Chi-** **Square**	***p*** **-value**	**Wald** **Chi-** **Square**	***p*** **-value**	**Wald** **Chi-** **Square**	***p*** **-value**	**Wald** **Chi-** **Square**	***p*** **-value**
Group	1	6.0	**0.014**	3.6	0.056	1.7	0.198	5.2	**0.022**	2.3	0.129	4.1	**0.042**
Sex	1	1.5	0.225	12.4	**<0.001**	15.8	**<0.001**	2.8	0.095	13.7	**<0.001**	17.3	**<0.001**
Side	1	0.1	0.708	0.0	0.902	0.1	0.771	1.4	0.244	1.0	0.314	0.1	0.790
Group-by-Sex	1	1.3	0.250	0.3	0.572	1.4	0.234	1.2	0.265	0.2	0.618	2.3	0.129
Group-by-Side	1	0.1	0.727	0.2	0.683	0.7	0.402	0.2	0.641	1.0	0.314	0.2	0.637
Group-by-Sex-by-Side	2	2.9	0.229	2.7	0.254	1.5	0.474	2.4	0.303	1.0	0.604	1.5	0.461

*df, degrees of freedom*.

*Bold text indicates significant differences (p < 0.05)*.

### Effect of Side

There was no main effect of side nor interaction involving side for any of the primary analyses (*p* ≥ 0.23). Therefore, for all figures, the sides were averaged together.

### Preferred and Prescribed Speeds

Our PAHD and Control groups did not differ in average preferred walking speed (Table 1). Although the average walking speed in the PAHD group was slightly lower than in the Control group, there was a wide range of preferred walking speeds in both groups (PAHD: 0.90–1.85 m/s; Control range: 0.89–1.65 m/s); no significant difference was noted (*p* = 0.879).

**Figure 3:**
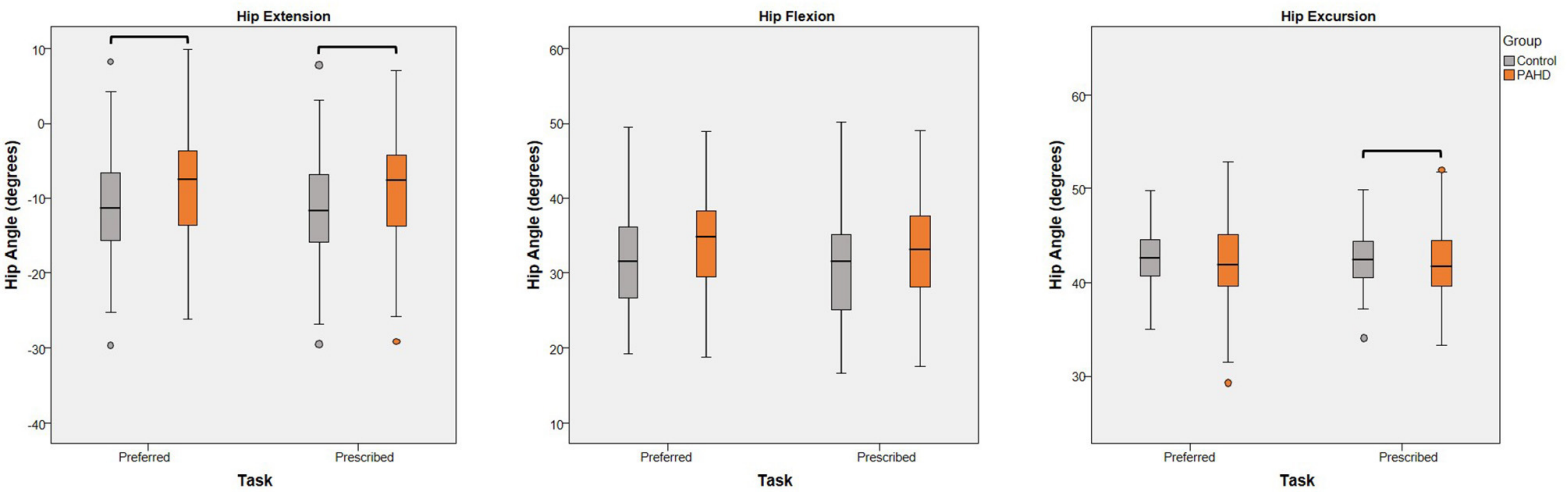
Effect of Group: Box plots (and outliers) for each dependent variable (peak hip extension, peak hip flexion, and hip excursion) for individuals with pre-arthritic hip disease (PAHD) and healthy individuals without pain (Control). Peak hip extension was less for individuals with PAHD than controls at both speeds. At the prescribed speed, individuals with PAHD walked with less hip excursion than controls. Bars indicate significant differences.

Despite walking at similar preferred speeds, the PAHD group walked with less peak hip extension than the Control group (mean difference: 2.9°; 95% Wald CI: 0.6, 5.1°; *p* = 0.014) confirming the expected finding (Table 3; Figure 3). No group difference was noted for excursion (mean difference: 0.8°; 95% CI: −0.4, 2.0; *p* = 0.198) or for peak hip flexion (mean difference: 2.1°; 95% CI: −0.05, 4.3; *p* = 0.056) despite slightly more flexion in the PAHD group.

At the prescribed walking speed of 1.25 m/s, the group difference for peak hip extension persisted and the difference for excursion became significant (Table 3). The PAHD group walked with 2.7° less hip extension (95% CI: 0.4, 5.0, *p* = 0.022) and 1.0° less hip excursion (95% CI: 0.04, 2.0; *p* = 0.042) compared to the Control group (Figure 3). Peak hip flexion, although slightly increased in the PAHD group (mean difference: 1.7°; 95% CI: −0.5, 3.8) was, again, not significantly different (*p* = 0.129).

### Structural Limitations

**Figure 4:**
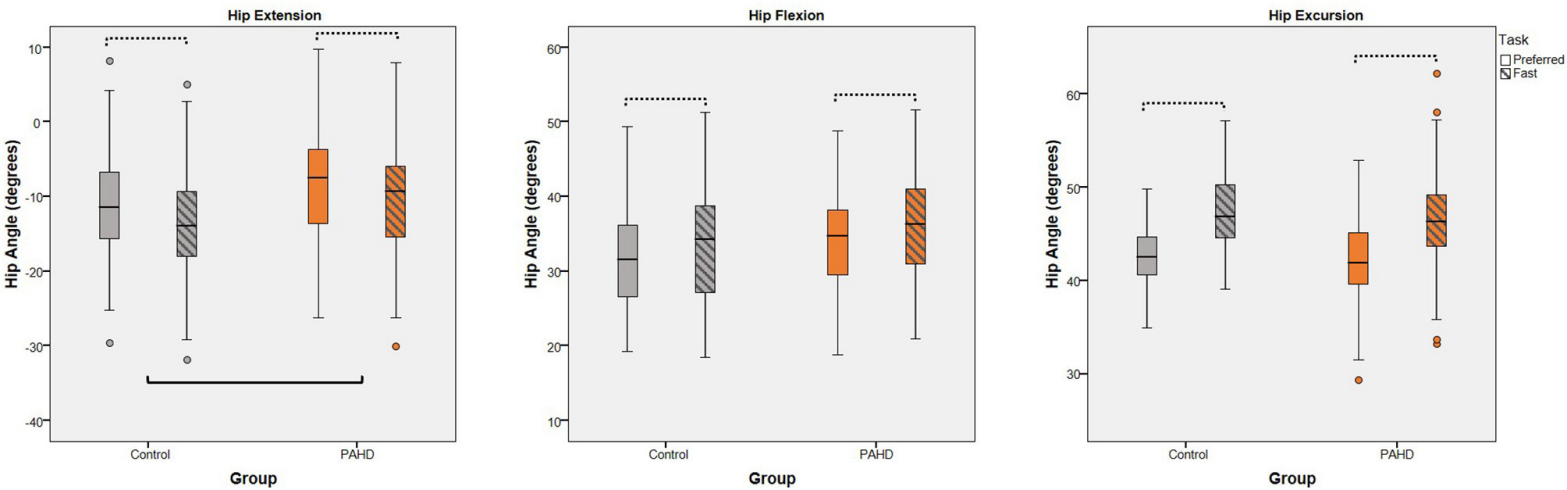
Interaction of Group and Task: Box plots (and outliers) for each dependent variable (peak hip extension, peak hip flexion, and hip excursion) for individuals with pre-arthritic hip disease (PAHD) and healthy individuals without pain (Control). All individuals, independent of group, increase hip angles at the fast speed compared to the preferred speed (dotted bars). The PAHD group had less hip extension than the Control group. No interaction of group and task was noted for any variable. Hip flexion is represented as positive. Solid bar indicates significant group differences.

To test if the reduction in hip extension was due to a structural limitation, we evaluated walking at a speed 25% faster than the preferred speed and assessed the *group-by-task* interaction. As expected, there was a main effect of walking task for each variable (*p* < 0.001) (Table 4). Peak hip extension, peak flexion, and hip excursion each were greater at the fast speed task compared to the preferred speed task, independent of group, demonstrating that our manipulation of gait speed was effective in eliciting increased hip motion (Figure 4). For peak hip extension, a main effect of group was also noted; the PAHD group had less peak hip extension than the Control group (mean difference: 3.2°; 95% CI: 0.8, 5.5; *p* = 0.008). However, the *group-by-task* interaction was not significant (*p* = 0.206), indicating that both groups similarly increased hip extension to walk at the faster speed. The PAHD group increased by an average 2.0° (95% CI: 1.3, 2.7) while the Control group increased by 2.6 (95% CI: 2.0, 3.1). A three-way interaction of *group-by-sex-by-task* was noted for peak hip flexion (*p* = 0.009); however, none of the within-sex pre-planned comparisons of interest were significant (*p* ≥ 0.118). For hip excursion, the *group-by-task* interaction was not significant (*p* = 0.058) despite a mean increase of 4.0° (95% CI: 3.4, 4.5) in the PAHD group and an increase of 4.6° (95% CI: 4.2, 5.1) in the Control group. Taken together, these results indicate that the PAHD group, on average, was able to increase their hip extension when walking at a faster speed, suggesting that the reduced hip extension noted at the preferred and prescribed walking speeds was not primarily due to structural limitation.

**Table 4:** Results of linear regression with generalized estimating equation correction for each dependent variable (hip extension, flexion, and excursion) modeling the interaction of group (PAHD, Control) and task (preferred speed, fast speed).

		**Group-by-Task**					
**Variable**		**Extension**		**Flexion**		**Excursion**	
	**df**	**Wald Chi-Square**	***p*** **-value**	**Wald Chi-Square**	***p*** **-value**	**Wald Chi-Square**	***p*** **-value**
Group	1	7.019	**0.008**	3.240	0.072	3.068	0.080
Sex	1	1.606	0.205	14.375	**<0.001**	18.354	**<0.001**
Side	1	0.054	0.816	0.049	0.824	0.000	0.984
Task	1	110.243	**<0.001**	140.505	**<0.001**	570.819	**<0.001**
Group-by-Task	1	1.600	0.206	0.147	0.701	3.587	0.058
Group-by-Sex-by-Task	2	0.784	0.676	9.475	**0.009**	3.205	0.201
Group-by-Sex	1	0.955	0.328	0.131	0.718	1.380	0.240
Group-by-Side	1	0.000	0.998	0.169	0.681	0.201	0.654
Group-by-Sex-by-Side	2	2.181	0.336	1.828	0.401	2.161	0.339
Group-by-Sex-by-Side-by-Task	4	2.026	0.731	0.787	0.940	1.992	0.737

*df, degrees of freedom*.

*Bold text indicates significant differences (p < 0.05)*.

### Pain

To test if the reduction in hip extension was likely a compensation for pain, we assessed hip kinematics only in individuals with PAHD, and compared those who reported pain during the walking task to those who reported no pain during the task. We did not find differences in peak extension, peak flexion, or excursion between the pain groups during the preferred and prescribed walking speed tasks (Table 5). We also did not find an effect of pain group when including the preferred and fast tasks, and their interaction, in the model (Table 6). Both groups were able to increase peak hip extension when walking at the faster speed compared to their preferred speed. There was a four-way interaction (*PainGroup-by-Sex-by-Side-by-Task)* for hip excursion (*p* = 0.030). When males and females were analyzed separately, again, no main effect of pain group or interaction including pain group was noted. In each of these analyses, we continued to find a main effect of sex; females walked with more hip flexion and excursion than males. Peak hip extension was not different between females and males in either analysis (*p* ≥ 0.642).

**Table 5:** Results of linear regression with generalized estimating equation correction for each dependent variable (hip extension, flexion, and excursion) limited to individuals with PAHD modeling PainGroup (pain with walking, no pain with walking) at the self-selected preferred walking speed and the prescribed (1.25 m/s) walking speed.

		**Preferred**						**Prescribed**					
**Variable**		**Extension**		**Flexion**		**Excursion**		**Extension**		**Flexion**		**Excursion**	
	**df**	**Wald** **Chi-** **Square**	***p*** **-value**	**Wald** **Chi-** **Square**	***p*** **-value**	**Wald** **Chi-** **Square**	***p*** **-value**	**Wald** **Chi-** **Square**	***p*** **-value**	**Wald** **Chi-** **Square**	***p*** **-value**	**Wald** **Chi-** **Square**	***p*** **-value**
PainGroup	1	0.1	0.752	0.3	0.561	1.5	0.228	0.0	0.897	0.0	0.835	0.0	0.902
Sex	1	0.0	0.901	7.4	**0.007**	14.1	**<0.001**	0.2	0.642	7.3	**0.007**	17.2	**<0.001**
Side	1	0.2	0.655	0.1	0.738	0.6	0.422	0.4	0.538	0.0	0.939	0.5	0.488
PainGroup-by-Sex	1	2.4	0.125	0.9	0.353	0.8	0.361	1.3	0.260	0.8	0.375	0.5	0.494
PainGroup-by-Side	1	0.5	0.496	1.5	0.228	3.4	0.063	0.8	0.379	0.2	0.641	1.6	0.207
PainGroup-by-Sex-by-Side	2	0.4	0.837	0.5	0.776	0.8	0.675	0.1	0.935	0.8	0.662	0.9	0.642

*Bold text indicates significant differences (p < 0.05)*.

*df, degrees of freedom*.

**Table 6:** Results of linear regression with generalized estimating equation correction for each dependent variable (hip extension, flexion, and excursion) in individuals with PAHD modeling the interaction of PainGroup (pain with walking, no pain with walking) and Task (preferred speed, fast speed).

		**Group-by-Task**					
**Variable**		**Extension**		**Flexion**		**Excursion**	
	**df**	**Wald Chi-Square**	***p*** **-value**	**Wald Chi-Square**	***p*** **-value**	**Wald Chi-Square**	***p*** **-value**
PainGroup	1	0.0	0.928	0.4	0.552	0.5	0.485
Sex	1	0.0	0.855	10.4	**0.001**	17.7	**<0.001**
StatSide	1	0.0	0.896	0.0	0.829	0.1	0.727
Task	1	17.8	**<0.001**	23.3	**<0.001**	124.6	**<0.001**
PainGroup-by-Task	1	1.0	0.326	0.0	0.949	1.8	0.185
PainGroup-by-Sex-by-Task	2	5.9	0.053	5.3	0.072	3.1	0.210
PainGroup-by-Sex	1	0.3	0.557	0.2	0.694	0.1	0.789
PainGroup-by-Side	1	0.1	0.774	1.8	0.182	0.8	0.377
PainGroup-by-Sex-by-Side	2	1.0	0.606	1.1	0.587	1.8	0.398
PainGroup-by-Sex-by-Side-by-Task	4	7.6	0.108	1.2	0.885	10.7	**0.030**

*Bold text indicates significant differences (p < 0.05)*.

### Sensitivity Analysis

We conducted three sensitivity analyses to understand if findings were affected by our inclusion criteria and pain thresholds. First, we limited the PAHD group to individuals with physician-diagnosed dysplasia, FAI syndrome, or acetabular labral tear, the three categories recommended for classification of hip-related pain in young and middle-aged adults (Reiman et al., 2020). We repeated the analyses in this limited dataset. The findings of decreased hip extension in the PAHD group compared to the Control group, and lack of *group-by-task* interaction persisted. Second, we limited the PAHD group to individuals who had not had previous hip surgery, and repeated the analyses. Again, the findings of decreased hip extension in the PAHD group compared to the Control group, and lack of *group-by-task* interaction persisted. Third, we assessed different thresholds for pain when subgrouping individuals in the PAHD group. Our original analysis separated individuals based on the report of *any* pain (average NRS > 0) during the walking task. We additionally tested using a NRS threshold of 1.0 and of 1.2 (the average pain rating within the PAHD group). Consistent with our primary results, we continued to find that, within the PAHD group, the peak hip extension from groups of individuals reporting more pain were not different compared to individuals reporting less pain.

### Time Series Analysis

**Figure 5:**
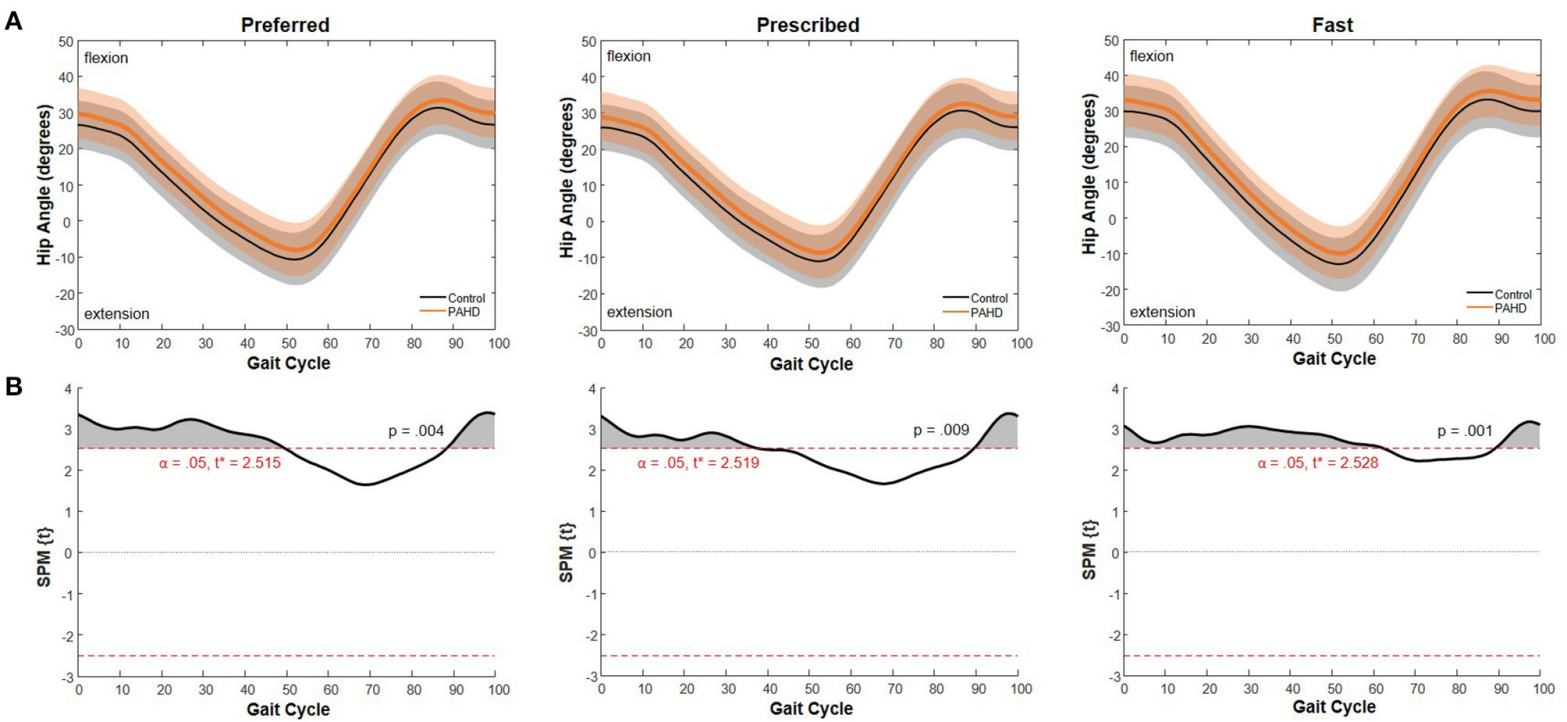
Sagittal Plane Hip Angle: **(A)** Mean hip angle during walking at a preferred speed for individuals with pre-arthritic hip pain (PAHD group, orange) and healthy individuals without pain (Control group, black). Shading represents one standard deviation above and below the mean. **(B)** Difference between the two mean curves at each 1% of the gait cycle (SPM{t}) (black), and threshold for significance (dashed line) using a general linear model with sex and walking speed as covariates. Shading indicates supra-threshold clusters where the difference is considered statistically significant.

The difference between the curve for the PAHD group and the curve for the Control group exceeded the critical threshold for the gait cycle except from 49.8% to 88.3% of the gait cycle for the preferred speed (Figure 5). The probability that a cluster of this size would occur by random chance was *p* = 0.004. At each point, the hip was in more flexion (or less extension) in the PAHD group than in the Control group (Figure 5). Similar results were noted for the prescribed and fast speeds. For the prescribed speed, the difference exceeded the critical threshold except from 38.4% to 89.3% of the gait cycle (*p* = 0.009). For the fast speed, the difference exceeded the threshold except from 62.2% to 89.3% (*p* = 0.001).

## Discussion

The aim of this study was to investigate sagittal plane hip kinematics during walking in individuals with pre-arthritic hip disease (PAHD) compared to individuals without any pain, relating any observed differences to changes common in individuals with established hip OA. Overall, the PAHD group walked with less peak hip extension than the Control group at all walking speeds. Peak hip flexion was not different between groups at any speed; hip excursion was less in the PAHD group than in the Control group at the fast and prescribed speeds. We noted that peak hip angles and excursion increased between walking at a preferred speed and a speed 25% faster than preferred. No interaction of group and walking task was noted, which was inconsistent with our hypothesis that individuals with PAHD would not increase hip extension as much as the Control group. Contrary to our hypothesis, within our PAHD group, we did not find a difference in hip angles or excursion between individuals who reported hip pain while walking and individuals who reported no pain during walking. Taken together, the results of this study indicate that kinematic alterations common in individuals with hip OA exist early in the continuum of hip disease and are not explained by walking speed, structural limitation, or current pain.

### Speed

Unlike studies of individuals with established hip OA (Constantinou et al., 2014, 2017) and some studies of individuals with PAHD (Hunt et al., 2013; Jacobsen et al., 2013), we did not observe a significant difference between groups for preferred walking speed. Both our PAHD and our Control groups, however, did have a wide array of preferred walking speeds, ranging from 0.85 m/s up to 1.85 m/s. As walking speed substantially affects observed kinematics and kinetics, monitoring walking speed when assessing for kinematic and kinetic differences is essential.

### Decreased Hip Extension and PAHD

Our finding of decreased hip extension during walking is consistent with separate studies of individuals with FAI syndrome (King et al., 2018; Lewis et al., 2018a), dysplasia (Endo et al., 2003; Jacobsen et al., 2013; Skalshøi et al., 2015), or hip OA (Ornetti et al., 2011; Eitzen et al., 2012; Rutherford et al., 2015; Constantinou et al., 2017; Farkas et al., 2019). Most of these studies were conducted at a self-selected walking speed that may be slower (whether significant or not) in the patient group than in the Control group. Similar to those studies, we found a decrease in peak hip extension in our PAHD group compared to controls when walking at their preferred speed. Additionally, we noted that hip extension continued to be reduced in the PAHD group even when all individuals walked at the same prescribed speed. Thus, the noted reduction in hip extension is not simply due to walking speed.

While differences in peak hip extension, but not flexion, were significant when evaluating only discrete datapoints, a visualization of the entire gait cycle and the time series analysis using SPM suggests a shift of the operating range toward flexion for the entire gait cycle. At each point, the hip was in more flexion (or less extension) in the PAHD group than in the Control group. This shift toward flexion was noted for each speed task from initial contact (0%) through midstance phase (38%) of the gait cycle, and again at terminal swing / deceleration (89%−100%). The differences extended further into stance phase in the preferred and fast trials (49.8% and 62.2%, respectively) than in the prescribed trial.

Walking with greater hip flexion at early stance has been associated with an increased risk of structural progression of hip OA (Kumar et al., 2018). Thus, these early kinematic changes may put individuals at increased risk for hip OA. The difference between the findings using peak angles and using SPM highlights limitations both in discrete statistics and in time series analyses. Analysis of discrete angles did not detect the difference in peak hip flexion when including the whole gait cycle, and thus, is highly influenced by the event or phase during which the discrete value is obtained. SPM, on the other hand, did not detect a difference between the curves around the time of peak hip extension for the preferred and prescribed speed tasks, likely due to variability in timing of that peak (range from 49–58% of the gait cycle). Selecting the appropriate analysis technique is a critical decision for all research.

### Structural Limitation

When walking at a faster than preferred speed, individuals with PAHD increased their peak hip extension compared to the preferred speed. The increase in peak hip extension demonstrates the ability to walk with more hip extension in this pre-hip OA population. This finding again suggests that the differences observed in individuals with PAHD are due to preferred or habitual movement patterns, and not simply structural limitations. Once severe hip OA is established, structural limitations may play a larger role in altered mechanics. Until that point, the reduced hip extension observed at preferred walking speeds appears to be modifiable.

While we compared hip mechanics between the preferred and faster walking speeds to understand structural limitations, analyzing how individuals achieve the faster speed could provide insight into appropriate interventions. For example, Tateuchi and colleagues analyzed cadence and stride length when individuals walked at a preferred speed compared to a fast speed (Tateuchi et al., 2021). They categorized individuals based on whether they increased their cadence, increased their stride length, or increased both to attain the faster walking speed. With this subgrouping on strategy, they found that individuals who increased cadence had better physical function scores than those who primarily increased stride length.

### Pain

While the common presumption is that individuals may decrease their hip extension during walking to reduce pain, we did not find a difference within our PAHD group between those reporting pain during the walking task and those reporting no pain during the task. The lack of difference persisted in sensitivity analyses of different pain thresholds. We acknowledge that in cases of severe pain, individuals tend to walk asymmetrically with a limp and substantial reductions in both hip excursion and peak hip extension are commonly noted. However, in the pre-arthritic population with less severe pain levels, the effect of pain is less straightforward, and it is unclear if altered movement contributes to or results from pain. It is possible that two groups exist: one that has pain because of the peak extension they use during walking and one that has pain despite already decreasing peak hip extension. When averaged together as a group, such distinctions would not be detectable and would obfuscate any pertinent findings. Further investigation of a larger cohort that allows for sub-classification based on peak hip extension and pain may be informative.

### Limitations

As with all studies, there were limitations to our approach. We did not include objective measures of available range of motion or strength in this analysis, as would be recommended by Mosler et al. (2020). However, the increase in peak hip extension at the faster walking speed suggests that individuals were not at the end of their available motion when walking at their preferred speed. In young active individuals, as included in our study, it is unlikely that kinematic alterations observed were due to muscle weakness. We did not randomize our walking speed tasks; we began with their self-selected preferred speed to capture their natural walking pattern, followed by the fast speed, and finally the prescribed speed. Given the short walking time (<10 min), it is unlikely that alterations observed were due fatigue. We did include a wide range of ages and used a convenience sample for our Control group; this resulted in our PAHD group being older than our Control group. As minimal changes in gait with age are noted in asymptomatic individuals (Rowe et al., 2021), it is unlikely that our findings were due to differences in group age. We included individuals who continued to have pain following surgical intervention—a group often excluded from analysis on the presumption that surgery would change movement patterns. Our finding did not change when excluding these individuals, suggesting that individuals with pain following hip surgery continue to have altered walking mechanics similar to those without surgery. This study was conducted as a cross-sectional study analyzing walking mechanics during a single session. A longitudinal study would be enlightening to see how group differences change with time.

## Conclusion

Based on results of this study of walking mechanics in 137 individuals with PAHD and 60 control participants, individuals with pre-arthritic hip pain modify their hip kinematics during walking; the most consistent modification was decreased peak hip extension. This modification was present at both preferred and prescribed walking speeds, indicating that it is not a result of slower walking speeds in those with hip pain. The modification was present despite having the *ability* to walk with more hip extension, as demonstrated by the increased hip extension at the faster walk speed. Efforts to address available range of motion without movement modification training will likely fail to change the observed movement patterns, and gains in available motion will fail to be maintained if not incorporated into daily activities. The modification was present despite not currently experiencing pain; thus, addressing pain is unlikely to normalize their movement patterns. Finally, it is not yet clear if these movement modifications are adaptive, and thus protective and should be encouraged, or if the movement modifications are maladaptive and destructive, and thus should be modified. It is clear that the decreased hip extension during walking noted in individuals with established hip OA is present far earlier in the disease process than anticipated.

## Data Availability Statement

The raw data supporting the conclusions of this article will be made available by the authors, without undue reservation.

## Ethics Statement

The studies involving human participants were reviewed and approved by Boston University Institutional Review Board. Written informed consent to participate in this study was provided by the participants or their legal guardian.

## Author Contributions

CL, AK, KL, AH, ZP, and EK contributed to the conception and design of the included studies. Data acquisition, analysis, and interpretation was done by CL, AH, KG, ZP, EK, AK, and KL. CL led the first draft of the manuscript, with HB, KG, EK, and KL contributing sections of the manuscript. All authors contributed to the manuscript revisions, and read and approved the submitted version.

## Conflict of Interest

The authors declare that the research was conducted in the absence of any commercial or financial relationships that could be construed as a potential conflict of interest.

## Publisher's Note

All claims expressed in this article are solely those of the authors and do not necessarily represent those of their affiliated organizations, or those of the publisher, the editors and the reviewers. Any product that may be evaluated in this article, or claim that may be made by its manufacturer, is not guaranteed or endorsed by the publisher.
